# Primary results of the VICTORION-NOVEL (LDL-C management Program in atherosclerotic cardiovascular disease patients with elevated LDL-C) lipid optimization multicenter implementation trial

**DOI:** 10.1016/j.ajpc.2026.101748

**Published:** 2026-07-16

**Authors:** Muthiah Vaduganathan, Alan Brown, Stacy L. Farr, Rena Moon, Ning Rosenthal, Batul Dahodwala, Michael D. Shapiro

**Affiliations:** a Brigham and Women’s Hospital, Boston, MA, USA; b Harvard Medical School, Boston, MA, USA; c Advocate Health Care, Oak Brook, IL, USA; d University of Missouri-Kansas City, Kansas City, MO, USA; e Saint Luke’s Hospital, BJC Health, Kansas City, MO, USA; f Premier Inc, Charlotte, NC, USA; g Novartis Pharmaceutical Corporation, East Hanover, NJ, USA; h Wake Forest University School of Medicine, Winston-Salem, NC, USA

**Keywords:** Statins, LDL-cholesterol, Lipid lowering treatment, Atherosclerotic cardiovascular disease

## Abstract

**Background:**

Many patients with atherosclerotic cardiovascular disease (ASCVD) have suboptimal longitudinal low-density-lipoprotein cholesterol (LDL-C) control. New system-level implementation strategies are needed to improve lipid optimization among high-risk adults.

**Methods:**

VICTORION-NOVEL was a prospective, multicenter implementation trial including 66 clinicians across ambulatory practices at 6 sites in the United States (9/5/2023–8/31/2024). Adults ≥18 years with established ASCVD, statin therapy and LDL-C ≥ 70 mg/dl were included. The intervention combined online dashboard for lipid management-related performance, peer-to-peer discussion calls and optional clinician resources and educational modules. Primary outcome was the probability of experiencing a composite endpoint defined as 1) achieving LDL-C < 70 mg/dl, 2) achieving ≥20% reduction in LDL-C, or 3) experiencing intensification of lipid lowering therapy (LLT) within 1 year.

**Results:**

Overall, 1097 patients (364 at 3 intervention sites, 733 at 3 usual care sites) were included (mean age 68, 45% women). At 1-year, similar proportion of patients (intervention: 44.2%, control: 48.2%, p = 0.22) experienced the composite endpoint. However, a significant time-varying effect on the primary outcome was observed with no benefit or lipid optimization favoring the control sites in the initial period (baseline to day 49 and day 50–218) with potentially late beneficial effects emerging thereafter (adjusted HR 1.34; 95% CI 1.07–1.68). At 1-year, a higher proportion of intervention patients achieved LDL-C < 70 mg/dl than control patients (22.5% vs. 16.2%, p < 0.001).

**Conclusions:**

The multi-faceted intervention was associated with a higher proportion of patients achieving LDL-C goals. The delayed emergence of benefit suggests that a lead-in period may be required for full effectiveness of similar system-level implementation efforts.

## 1 Introduction

Among high-risk patients with atherosclerotic cardiovascular disease (ASCVD), contemporary multi-specialty guidelines strongly recommend lipid optimization, specifically targeting low-density-lipoprotein cholesterol (LDL-C), as a core facet of cardiovascular risk reduction [1]. Statins are prioritized as foundational therapy in guidelines, but among individuals with ASCVD with residually elevated LDL-C levels greater than 70 mg/dl, consideration of additional non-statin therapies is suggested, such as advanced lipid lowering therapies (LLTs) [1]. Despite the availability of multiple LLT options, > 90% of patients with ASCVD do not progress beyond initial statin therapy and fail to achieve the guideline recommended LDL-C targets [2]. Only approximately 20% of patients with ASCVD who receive statin therapy alone achieve LDL-C goals of < 70mg/dl [3,4]. Simulation models using a large medical and pharmacy claims database estimated that, with appropriate intensification of LLT, over 99% of patients with ASCVD could have achieved the LDL-C goal compared to 25% before intensification [5].

New system-level implementation strategies are needed to improve lipid optimization among high-risk adults. Provision of tailored patient data at clinician’s fingertips using a performance monitoring and educational platform to encourage clinician and patient engagement may facilitate more optimal lipid management at scale. We conducted the VICTORION-NOVEL (LDL-C Management Program in Atherosclerotic Cardiovascular Disease Patients with Elevated LDL-C) trial to evaluate the effectiveness of a multi-faceted implementation program that combines online dashboard for lipid management-related performance, peer-to-peer discussion calls to facilitate improvement through reflection, self-assessment, and feedback on intervention components, and optional educational clinician resources (“the intervention”) on improving LDL-C optimization among patients with ASCVD.

## 2 Methods

### 2.1 Design of VICTORION-NOVEL

VICTORION-NOVEL was a prospective, multicenter quasi-experimental, cluster-assigned implementation trial including 66 clinicians across ambulatory practices at 6 sites in the United States conducted between September 2023 and August 2024. As the implementation online dashboard required direct electronic health record (EHR) access, sites were approached for willingness and capacity to share EHR data components to be integrated into the dashboard. Six sites (5 non-academic, 1 academic health system) that had the capacity for EHR platform integration were assigned to intervention arm (3 sites) or usual care / control arm (3 sites). Because clinicians within the same health system were likely to interact and potentially share clinical care teams (such as nursing, care coordination, and pharmacy staff) and patients, a cluster-based study design was selected at the site level and all clinics within each site followed the same intervention or control assignment. Geographically diverse health systems were selected to participate in the study to ensure that clinicians and patients were only included in either the intervention or control arm. Because intact sites (clusters), rather than individual patients, were assigned to study arms, and because assignment was not randomized, the trial is quasi-experimental rather than an individual-level randomized trial.

At participating sites, all adults ≥18 years with established ASCVD, who were already treated with any dose of statin therapy, and with LDL-C ≥ 70 mg/dl between August 1, 2022, and July 31, 2023 (12-month baseline period) were included. Evidence of ASCVD was defined using International Classification of Diseases, 10th Edition (ICD-10) diagnoses and procedure codes for: 1) myocardial infarction (MI), ischemic stroke or transient ischemic attack (TIA), peripheral artery disease (PAD), ischemic heart disease, coronary heart disease, or unstable angina, or 2) coronary revascularization including percutaneous coronary intervention (PCI) and coronary artery bypass graft (CABG). The date the online dashboard became available to all three intervention sites was considered as “index” date (September 5, 2023) for all patients. Patients were excluded if they were: 1) without a valid LDL-C measurement; 2) with active cancer (defined as receiving chemotherapy, radiation, or surgery for cancer); 3) actively enrolled in hospice care; or 4) with LLT intensification (defined as switching to a high-intensity statin or addition of a non-statin LLT) or LLT change during baseline period.

### 2.2 Multi-faceted lipid optimization implementation intervention

The following components of the multi-faceted program were only made available to sites assigned to the intervention arm (Fig. 1):

• Access to an online dashboard (Supplement Figure S1)
• Three live peer-to-peer (P2P) discussion calls
• (Optional) Online resources for clinicians
• (Optional) Populated 3Is (Identification, Intervention, and Interaction) Framework™
• (Optional) Educational modules for clinicians related to the use of the online dashboard and how to engage patients to address barriers and challenges to managing their LDL-C
• One focus group call for each intervention site towards the end of the study to collect feedback on platform, tools, and overall study delivery

**Fig. 1:**
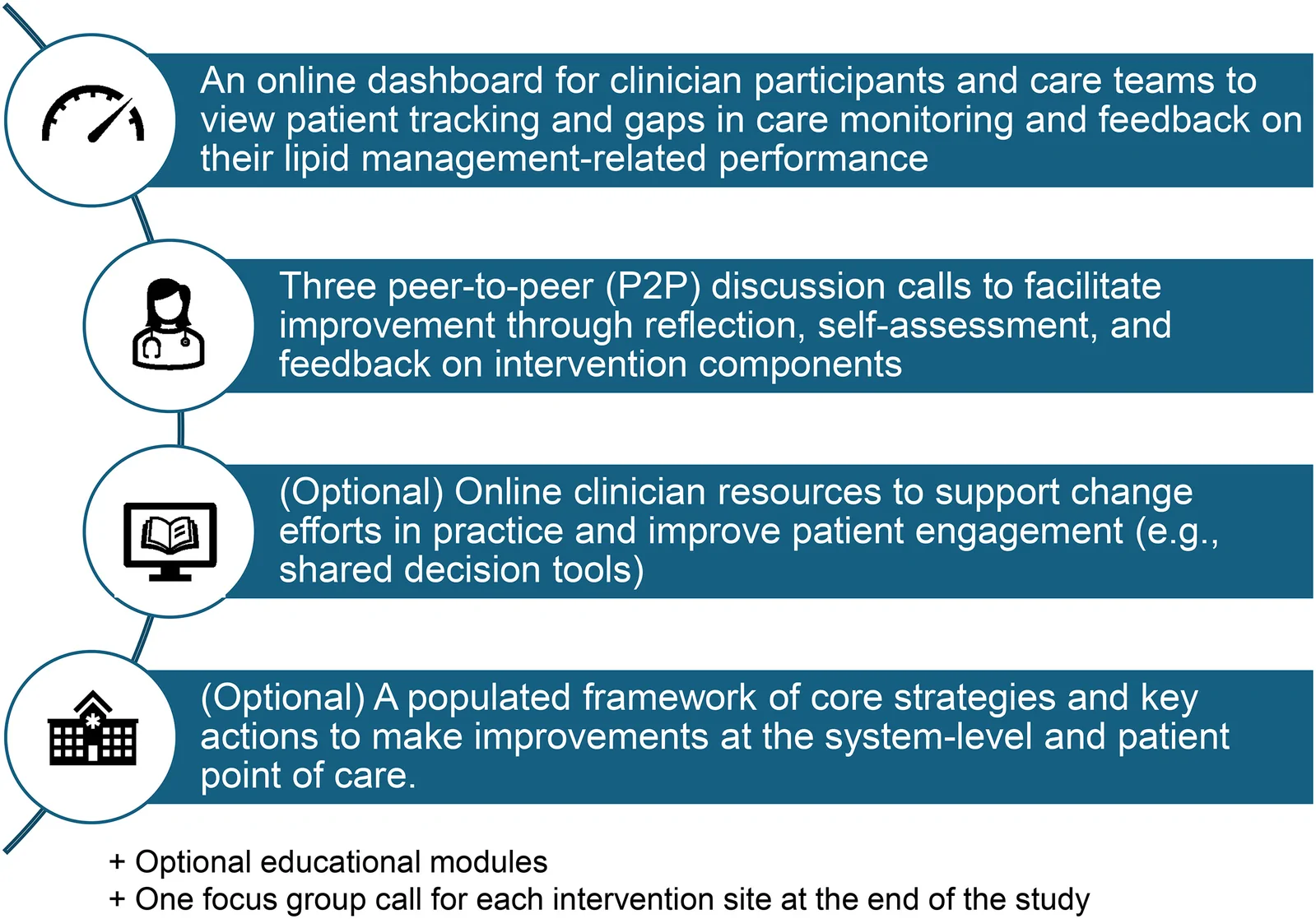
**Summary of the multi-faceted program (intervention)**.

Participating clinicians were required to be prescribers (physicians or advance practice providers), but access to the intervention was granted to their broader clinical teams at intervention sites including nurses, care coordinators, and pharmacists. The intervention lasted for a period of 12 months at the 3 intervention sites. At the end of the 12-month intervention, the 3 control sites were provided access to the online dashboard with their respective patients’ data during the intervention period and the resources for 3 months. The multi-faceted implementation study was approved by the institutional review board (IRB) and included quantitative and qualitative data components.

### 2.3 Study outcomes

The prespecified primary outcome was a composite lipid optimization endpoint defined as 1) achieving LDL-C < 70 mg/dl; or 2) experiencing 20% reduction in LDL-C from baseline; or 3) having LLT intensification between September 6, 2023, and August 31, 2024 (12-month follow-up period). The goal of three components of the composite endpoint was to capture complementary dimensions of lipid optimization that an implementation program is intended to influence: attainment of LDL-C < 70 mg/dl reflects achievement of the guideline-recommended treatment target; a ≥ 20% reduction in LDL-C reflects a clinically meaningful response; and LLT intensification reflects the process change (clinician action). A patient was counted as meeting the composite if any one of the three components was satisfied; the components were therefore weighted equally, with none required and none given numerical priority. Secondary outcomes included individual components of the primary outcome (proportions of patients achieving LDL-C < 70 mg/dl, achieving ≥ 20% reduction in LDL-C, and with LLT intensification), proportion of patients with LDL-C testing, and absolute and percent changes in LDL-C during 12-month follow-up period. Secondary outcomes also included qualitative assessments of barriers and facilitators to LDL-C management and clinicians’ perceived success of the intervention.

### 2.4 Statistical analysis

Descriptive statistics were used to present baseline patient and hospital characteristics of included patients across the 6 participating sites. Continuous variables were reported as mean (standard deviation) and median (1st quartile, 3rd quartile), and categorical variables were reported as counts and percentages. For unadjusted comparisons between intervention and control arms, two sample *t*-tests were used for continuous measures and Pearson’s χ^2^ tests or Fisher’s exact tests were used for categorical measures. For the time to event adjusted analysis of primary outcome, extended Cox regression models were used because the main exposure variable violated the proportional hazard assumption. Therefore, the follow-up period was segmented using single Heaviside functions with cut-points at 50 and 219 days post-index that resulted in time-segmented hazard ratios. These cut-points were not prespecified; they were selected post hoc based on the timing of the peer-to-peer calls and on inspection of the Kaplan-Meier curves. The primary analysis included the 1097 patients with at least one office visit or LDL-C test during follow-up. To assess whether excluding the 178 enrolled patients without follow-up data affected the intention-to-treat interpretation of the primary outcome, we conducted a conservative sensitivity analysis that retained all 1275 enrolled patients; patients without a follow-up were classified as not achieving the composite endpoint, and time-to-event models censored such patients on day 1 of follow-up. Adjusted odds of secondary outcomes were assessed using multivariable logistic regression models. Given the limited number of clusters (sites) and the possibility of baseline imbalance in included participants across intervention vs. control/usual care groups, all primary and secondary analyses were adjusted for a prespecified set of clinical covariates. Model covariates, selected a priori based on clinical relevance, were age, sex, race, ethnicity, hypertension, atrial fibrillation, Charlson Comorbidity Index score category, and ASCVD type. Final model covariates were selected using the backward selection method, with a significance level of p < 0.10 for covariates to stay in the model. For all primary and secondary outcome models, robust standard errors were applied to adjust for clustering of patients within sites. All analyses and figures were performed and generated using R v.3.6.3 or higher (R Foundation for Statistical Computing, Vienna, Austria).

### 2.5 Qualitative analysis

Three peer-to-peer calls (three intervention sites together) were completed during the intervention – the first in November 2023 to provide an overview of the online dashboard and to discuss the framework and interactive function of tools; the second in April 2024 to review and discuss current lipid management guidelines; and the third in July 2024 to obtain participant feedback and reflection on the study. Focus group calls (each of three intervention sites individually) were conducted in September and October 2024 to collect feedback on the online dashboard, tools, and overall perceptions related to the intervention. In addition, clinician surveys, conceptually informed by the Tailored Implementation in Chronic Disease (TICD) determinants framework [6], were administered pre- and post-intervention to all intervention and control arm clinicians to collect participating clinicians’ characteristics, beliefs, and perceived barriers and facilitators to LDL-C management.

## 3 Results

Site, clinician, and patient-level flows are summarized in CONSORT-style diagram (Supplement Figure S2).

### 3.1 Baseline characteristics

Of 1275 patient participants, 1097 had at least one office visit or LDL-C testing during the follow-up period (364 at intervention sites, 733 at control/usual care sites); 178 patients were considered lost to follow-up and excluded from the analysis. Baseline demographics and clinical characteristics were similar between included vs. excluded patients (Supplement Table S1). The percentage of patients lost to follow-up were similar in both arms (13% [56 out of 420] for intervention and 14% [122 out of 855] for control, p = 0.65)

Mean age was similar between the intervention and control arms (67.1 vs. 68.2 years, p = 0.13) (Table 1). Patients in the control arm were more likely to be White (88.5% vs. 79.1%, p < 0.01) and non-Hispanic (97.4% vs. 67.3%, p < 0.01) compared to patients in the intervention arm. A higher proportion of intervention arm patients had hypertension (80.8% vs. 47.1%, p < 0.01) and atrial fibrillation (20.1% vs. 2.0%, p < 0.01) than control arm patients. Baseline mean systolic blood pressure and body mass index (BMI) were similar between the two arms. Comorbid disease burden was moderate to high in this population with 73.8% having 1–3 comorbidities and 10.0% having 4 or more comorbidities. Patients in the intervention arm were more likely to have PAD (17.9% vs. 13.6%) or to have undergone a revascularization procedure (28.3% vs. 13.0%), and less likely to have acute MI or other ischemic heart diseases (8.8% vs. 33.3%) or ischemic stroke (24.7% vs. 28.9%) compared to patients in the control arm (all p < 0.01, Table 1). Distribution of LLT treatment regimen was similar between the 2 groups, with statin alone being the most common regimen (88.6%) followed by statin + ezetimibe (8.9%) and statin + a proprotein convertase subtilisin/kexin type 9 inhibitor (PCSK9i, 1.4%).

**Table 1:** Baseline characteristics.

	Overall		Intervention		Control		p-values
	(N = 1097)		(N = 364)		(N = 733)		
**Age Category, n(%)**							
18–39 years	7	0.6%	2	0.5%	5	0.7%	0.43
40–59 years	230	21.0%	79	21.7%	151	20.6%	
60–74 years	542	49.4%	189	51.9%	353	48.2%	
75+ years	318	29.0%	94	25.8%	224	30.6%	
**Age, continuous, in years**							
Mean-Std Dev	67.83	10.97	67.12	10.41	68.18	11.22	0.13
Median (Q1-Q3)	68.00	(61.00- 76.00)	68.00	(61.00- 75.00)	69.00	(61.00- 76.00)	
**Female sex, n(%)**	493	44.9%	154	42.3%	339	46.2%	0.22
**Race, n(%)**							
White	937	85.4%	288	79.1%	649	88.5%	<0.001
Black	79	7.2%	39	10.7%	40	5.5%	
Other	81	7.4%	37	10.2%	44	6.0%	
**Ethnicity, n(%)**							
Hispanic	27	2.5%	13	3.6%	14	1.9%	< 0.001
Non-Hispanic	959	87.4%	245	67.3%	714	97.4%	
Unknown	111	10.1%	106	29.1%	5	0.7%	
**Primary insurance payer, n(%)**							
Medicare	225	20.5%	57	15.7%	168	22.9%	<0.01
Medicaid	8	0.7%	1	0.3%	7	1.0%	
Commercial Insurance	43	3.9%	13	3.6%	30	4.1%	
Uninsured	3	0.3%	3	0.8%	0	0.0%	
Other/Unknown	818	74.6%	290	79.7%	528	72.0%	
**Hypertension, n(%)**	639	58.2%	294	80.8%	345	47.1%	< 0.001
**Atrial fibrillation, n(%)**	88	8.0%	73	20.1%	15	2.0%	< 0.001
**Familial hypercholesterolemia, n(%)**	0	0.0%	0	0.0%	0	0.0%	
**Baseline Charlson comorbidities, n(%)**							
Myocardial infarction	252	23.0%	29	8.0%	223	30.4%	< 0.001
Heart failure	181	16.5%	76	20.9%	105	14.3%	< 0.01
Peripheral artery disease	270	24.6%	101	27.7%	169	23.1%	0.09
Cerebrovascular disease	417	38.0%	112	30.8%	305	41.6%	< 0.001
Dementia	7	0.6%	2	0.5%	5	0.7%	0.80
Chronic pulmonary disease	128	11.7%	20	5.5%	108	14.7%	< 0.001
Rheumatic disease	10	0.9%	1	0.3%	9	1.2%	0.12
Peptic ulcer disease	6	0.5%	2	0.5%	4	0.5%	0.99
Mild liver disease	11	1.0%	4	1.1%	7	1.0%	0.82
Diabetes	217	19.8%	59	16.2%	158	21.6%	0.04
Hemiplegia or paraplegia	5	0.5%	1	0.3%	4	0.5%	0.53
Renal disease	118	10.8%	21	5.8%	97	13.2%	< 0.001
Moderate or severe liver disease	1	0.1%	0	0.0%	1	0.1%	0.48
Any malignancy	51	4.6%	11	3.0%	40	5.5%	0.07
Metastatic solid tumor	1	0.1%	0	0.0%	1	0.1%	0.48
AIDS/HIV	1	0.1%	0	0.0%	1	0.1%	0.48
**Charlson Comorbidity Index (CCI) score category, n(%)**							
0	177	16.1%	105	28.8%	72	9.8%	< 0.001
1–3	810	73.8%	243	66.8%	567	77.4%	
4+	110	10.0%	16	4.4%	94	12.8%	
**Mean CCI (std. dev.)**	1.82	1.82	1.34	1.50	2.05	1.91	< 0.01
**Patients with an ASCVD event at baseline, n(%)**							
Acute MI or other ischemic heart diseases	276	25.2%	32	8.8%	244	33.3%	< 0.01
Ischemic stroke	302	27.5%	90	24.7%	212	28.9%	
TIA	43	3.9%	7	1.9%	36	4.9%	
PAD	165	15.0%	65	17.9%	100	13.6%	
Unstable angina	12	1.1%	0	0.0%	12	1.6%	
Revascularization procedure	198	18.0%	103	28.3%	95	13.0%	
**LLT treatment regimen, n(%)**							
Statin	972	88.6%	325	89.3%	647	88.3%	0.29
Ezetimibe	0	0.0%	0	0.0%	0	0.0%	
PCSK9i	0	0.0%	0	0.0%	0	0.0%	
Bempedoic acid	0	0.0%	0	0.0%	0	0.0%	
Inclisiran	0	0.0%	0	0.0%	0	0.0%	
Statins + ezetimibe	98	8.9%	35	9.6%	63	8.6%	
Statins + PCSK9i	15	1.4%	2	0.5%	13	1.8%	
Statins + bempedoic acid	3	0.3%	0	0.0%	3	0.4%	
Statins + inclisiran	0	0.0%	0	0.0%	0	0.0%	
Statin + 2 or more	9	0.8%	2	0.5%	7	1.0%	

### 3.2 Primary composite lipid optimization outcome

At 1-year, 44.2% in the intervention group and 48.2% in the control group experienced the composite lipid optimization endpoint (p = 0.22, Table 2). However, the proportional hazards assumption was violated such that significant time-varying effects were detected. Segmenting the follow-up period allowed for better characterization of the time-dependent effects and addressed the proportional hazards assumption. The probability of experiencing the composite endpoint between index date and 218 days post-index date was similar between the 2 groups (Fig. 2). However, on or after post-index day 219, the probability of experiencing the composite endpoint appeared to favor the intervention arm (hazard ratio [HR] 1.68; 95% confidence interval [CI] 1.08–2.60). After adjusting for race, ethnicity, and hypertension, the probability of experiencing the composite endpoint between post-index day 50–218 appeared higher in the control arm compared with the intervention arm (adjusted HR 0.54; 95% CI 0.37–0.80), while the probability of experiencing the composite endpoint on or after post-index day 219 remained higher for the intervention arm compared with the control arm (adjusted HR 1.34; 95% CI 1.07–1.68). Because the time-segment cut-points were selected post hoc rather than prespecified, these time-varying hazard ratios, and the statistical significance of the individual segments, should be interpreted as exploratory and hypothesis-generating.

**Table 2:** Primary and Secondary Outcomes.

	Overall		Intervention		Control		p-values
	(N = 1097)		(N = 364)		(N = 733)		
**Patients with composite endpoint, n(%)**	514	46.9%	161	44.2%	353	48.2%	0.22
**Absolute difference in LDL-C level between baseline and 12-month, mg/dl**							
N	728		224		504		0.01
Mean-Std Dev	−9	37	−14	33	−7	38	
Median (Q1-Q3)	−7	(−26- 7)	−11	(−29- 2)	−5	(−25- 10)	
**Percentage difference in LDL-C level between baseline and 12-month, %**							
N	728		224		504		< 0.01
Mean-Std Dev	−6	36	−11	31	−4	38	
Median (Q1-Q3)	−8	(−25- 9)	−12	(−31- 2)	−6	(−24- 11)	
**Patients achieving LDL-C goal (< 70mg/dl), n(%)**	201	18.3%	82	22.5%	119	16.2%	< 0.001
**Patients achieving 20% reduction in LDL-C, n(%)**	347	31.6%	110	30.2%	237	32.3%	0.06
**Patients with intensification of LLT, n(%)**	226	20.6%	63	17.3%	163	22.2%	0.06
**Patients with LDL-C testing, n(%)**	729	66.5%	225	61.8%	504	68.8%	0.02
**Absolute difference in non-HDL-C level between baseline and 12-month, mg/dl^1^**							
N	370		110		260		0.71
Mean-Std Dev	−8	42	−9	41	−7	42	
Median (Q1-Q3)	−7	(−28- 11)	−6	(−29- 7)	−8	(−27- 12)	
**Percentage difference in non-HDL-C level between baseline and 12-month, %**							
N	370		110		260		0.68
Mean-Std Dev	−4	31	−5	29	−4	31	
Median (Q1-Q3)	−6	(−24- 10)	−5	(−23- 6)	−7	(−24- 11)

**Fig. 2:**
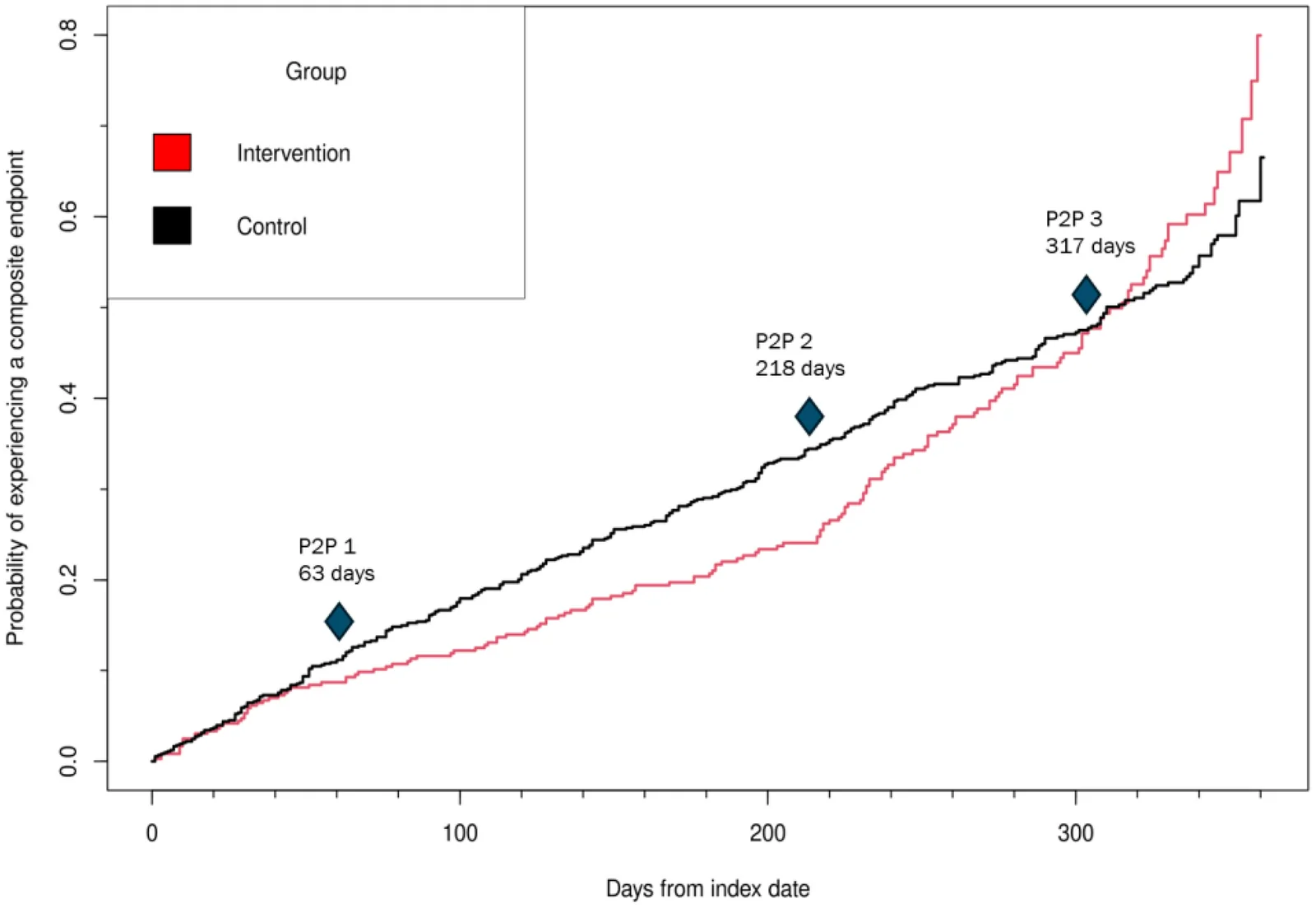
**Inverse Kaplan-Meier Curve of the Primary Composite Lipid Optimization Outcome, Relative to Timing of Peer-to-Peer Calls**. The primary outcome was the probability of experiencing a composite endpoint defined as 1) achieving LDL-C < 70 mg/dl, 2) achieving ≥ 20% reduction in LDL-C, or 3) experiencing intensification of lipid lowering therapy (LLT). Higher probability denotes better lipid optimization.

In a conservative intention-to-treat sensitivity analysis that retained all 1275 enrolled patients, the composite was met by 38.3% of intervention and 41.2% of control patients with unadjusted and adjusted probabilities of experiencing composite endpoint same or similar to those in the primary analysis (Supplement Table S2).

### 3.3 Secondary outcomes: quantitative

A higher proportion of patients in the intervention arm achieved LDL-C goal at 22.5% compared with patients in the control arm at 16.2% (p < 0.01). Similar proportions of patients achieved 20% reduction in LDL-C in both arms (30.2% vs. 32.3%, p = 0.06) while a lower proportion of patients experienced intensification of LLT in the intervention arm (17.3% vs. 22.2%, p = 0.06). The reduction in the mean LDL-C level between baseline and follow-up periods was higher in the intervention arm at 13.9 mg/dl (11.3% reduction) compared to the control arm at 6.7 mg/dl (3.6% reduction) (p = 0.01). Although the study aimed to collect apoB and Lp(a) measures, these were not routinely tested (only one patient with an apoB measure) and therefore could not be analyzed.

The probability of achieving LDL-C goal between index date and 218 days post-index date was similar between the two groups (adjusted HR 0.83; 95% CI 0.25–2.75, Supplement Figure S3a). On or after post-index day 219, the probability of achieving LDL-C goal was higher for intervention arm patients compared to those in the control arm, but the difference was not statistically significant (adjusted HR 3.23; 95% CI 0.84–12.45). The probability of achieving 20% reduction in LDL-C was also similar between the two groups (Supplement Figure S3b). The probability of LLT intensification was lower for intervention arm patients between post-index days 50 and 218, but the difference was not statistically significant (unadjusted HR 0.49, 95% CI 0.20–1.18, Supplement Figure S3c). On or after post-index day 219, the probability of LLT intensification was higher for intervention arm patients compared to those in the control arm, but the difference was not statistically significant (unadjusted HR 1.70; 95% CI 0.84–3.44). LLT intensification showed the biggest difference between the two groups prior to post-index day 219.

### 3.4 Secondary outcomes: qualitative

#### 3.4.1 Clinician survey results

Sixty-six clinicians completed the baseline clinician survey of barriers and facilitators at the start of the study, 40 in the intervention group and 26 in the control group. Of those who completed the baseline clinician survey, 37 (14 in the intervention group and 23 in the control group) also completed the post-intervention clinician survey. Most respondents held a Doctor of Medicine (MD) degree (69.7%), followed by an advanced registered nurse practitioner (ARNP) degree (22.7%), and many specialized in interventional cardiology (42.4%) and general cardiology (40.9%). A significantly higher proportion of clinicians in the control arm were practicing at hospital-based academic sites and suburban areas compared to clinicians in the intervention arm. Both intervention and control arm clinicians indicated that their patients did not have a good understanding of cholesterol and target LDL-C levels, understand what good and bad cholesterol were, or know that high cholesterol can be a silent killer. Primary barriers to taking medications for patients were things they heard or read and because they were concerned about side effects. Both intervention and control clinicians stated that they were comfortable with escalating LLT and believed that adding ezetimibe and other non-statin therapies (PCSK9i or small interfering RNA) to statin therapy was safe.

#### 3.4.2 Peer-to-Peer group and focus group discussion results

For the 3 P2P calls, 41 clinicians attended the first call, and 15 attended the third call (no attendance report was available for the second call). Thirteen clinicians participated in the 3 focus group calls, 6 in the first, 3 in the second, and 4 in the third (Supplement Table S3). Primary topics (Table 3) discussed during the P2P and focus group calls included the value and usefulness of the dashboard, the integration of the dashboard with practice workflow, the value of site comparison data, the value of the study, and the study design. Overall, participants said that the program was valuable because it was evidence-based, offered useful tools (dashboard, barrier survey), and raised awareness of patients’ LDL-C levels. The dashboard and P2P calls supported clinicians in more active decision-making, a preventive care focus, and peer learning. Participants also appreciated the site comparison data and patient education resources (success tool, medication tool).

**Table 3:** Select qualitative feedback collected from clinician-participants, reported by theme.

1. Value and Usefulness of study platform		
	Feedback	Role
**Facilitators**	*“It helped to have the overall visualization that the dashboard provided.”*	Nurse
	*“The physicians took advantage of the many educational materials available on the platform.”*	Cardiologist/Investigator
**Barriers**	*“I think it wasn't all perfectly intuitive on the platform.”*	Cardiologist
	“*It would be good if that [educational] information …was more accessible through our system.*”	Cardiologist

2. Integration of study dashboard with practice workflow		
	Feedback	Role
**Barriers**	*“It was cumbersome to go through the platform to print out the patient resources.”*	Cardiologist
	*“…Didn't really flow with our workflow very well.”*	Nurse

3. Value of practice site comparison data		
	Feedback	Role
**Facilitators**	“It was helpful to see how our site was doing in comparison to the other sites.”	Nurse/ Research Coordinator
	*“That's one of the things I always think is useful to know – how we compared to other sites.”*	Cardiologist/Investigator
	*“I've always thought that a really good report card to individual doctors would shift their pride a little bit, like you are doing…with this study.”*	Cardiologist

4. Value of the overall study		
	Feedback	Role
**Facilitators**	*“[Evaluating] barriers gave me a lot of ideas, maybe that I have not thought about before.”*	Cardiologist
	*“We can use all the tools for the improvement of our patients. I really appreciate being a part of this [study]*.”	Cardiologist
	“*They [the tools and resources] helped me apply information to my practice*.”	Cardiologist
	*“I think the [patient-specific tools] were very important to us. I think it empowers the patients.”*	Cardiologist

5. Overall study design		
	Feedback	Role
**Facilitators**	*“The program is evidence-based, and it’s very good.”*	Cardiologist
	*“Really good information from [peer-to-peer sessions] … definitely helped me apply that information to my practice.”*	Cardiologist
	*“I think the evaluating barriers [framework] was perhaps the most useful.”*	Cardiologist
**Barriers**	*“It was difficult for providers to commit to the educational program to make an impact.”*	Cardiologist/Investigator

Clinicians did experience some technical and workflow challenges (e.g., log-ins, limited EHR integration), which affected usability and adoption. Patient engagement was mixed, according to participants. Education tools were helpful, but surveys had low response rates, and myths about statins remained a barrier. Finally, overall practice impact was limited, but participants suggested future improvements such as stronger EHR integration and linking study interventions to patient outcomes.

## 4 Discussion

While the multi-faceted intervention tested in the VICTORION-NOVEL trial did not significantly improve the primary outcome of probability of achieving LDL-C goal, achieving 20% reduction in LDL-C, or intensifying LLT at 1-year, we did observe important time-varying effects such that there was potential delayed benefit seen in the latter part of the follow-up period. In addition, a higher proportion of patients achieved LDL-C goals and there was greater LDL-C lowering in patients managed at intervention sites compared to those at usual care sites. While the results were not statistically significant, the intensification of therapy in the intervention arm improved after post-index day 219 and might be one of the factors that helped in achieving LDL-C goal. Given the quasi-experimental, cluster-assigned design with only six clusters, the baseline imbalance between arms, and the post hoc nature of the time-segmented analyses, these findings are primarily hypothesis-generating about effectiveness and adoption.

VICTORION-NOVEL highlights some of the complexities of implementation of lipid management strategies in real world clinical care. The delayed emergence of benefit suggests that a lead-in period may be required for full effectiveness of similar system-level implementation efforts. A lead time may have been necessary for site on-boarding activities, time to gain familiarity with effective and efficient use of the online dashboard, dissemination of tools to all practicing clinicians (beyond the site champions that primarily led the trial locally at each health system). The potential reasons around the exact timing of the late benefit are uncertain but appear to correspond with the second peer-to-peer call (post-index day 218).

In a multi-center prospective observational study on the change in LLT use, Cannon et al. [7] reported that practice characteristics including teaching status and presence of lipid protocol were associated with LLT intensification after 2 years. We recognize that VICTORION-NOVEL was limited to only 6 selected sites that had the capacity to integrate the EHR-based implementation effort, so imbalances in site characteristics and practice settings may have contributed to the challenges in showing a robust benefit of the intervention. Notably, a higher proportion of patients at control sites had LDL-C testing or clinic visits during the study which might be explained by this site-based imbalance in care patterns.

Several findings warrant cautious interpretation. Although LDL-C goal attainment and mean LDL-C reduction were greater in the intervention arm, both LDL-C testing during follow-up (61.8% vs. 68.8%) and documented LLT intensification (17.3% vs. 22.2%) were lower in the intervention arm, even though we see time-varying effects through the study period (Table 2). This apparent discordance is more likely to reflect the baseline imbalance between arms, differences in the timing and frequency of LDL-C measurement, and site-level differences. Because lower testing in the intervention arm reduces ascertainment of both goal attainment and intensification, the direction of bias is uncertain. The qualitative findings help explain both the lower testing and the delayed emergence of benefit: clinicians described log-in difficulties, limited EHR integration, and workflow friction that impeded routine use of the dashboard early in the study.

Although most physicians indicated escalating statin therapies or adding non-statin therapies are safe, they indicated concerns with affordability of and adherence to multi-drug regimens for patients as well as challenges in follow-up testing and time constraints. During peer-to-peer and focus group discussions, participating clinicians stated that they appreciated the program providing helpful tools and resources. However, they indicated technical issues or being overwhelmed by processes as barriers to clinician uptake of the online dashboard and education program.

While the dashboard appears to be an important component for success, sustained engagement likely requires additional motivational mechanisms. These may include incentives or competitive elements, such as peer comparisons, benchmarking against national or local quality metrics, or alignment with financial or performance-based incentives. Ongoing reinforcement of the project’s importance is also essential to prevent reversion to prior practice patterns and to maintain engagement over time.

### 4.1 Study limitations

This study had several limitations. First, due to the range of clinic locations and populations receiving care at relatively few target health systems, there were imbalances at baseline in patients at intervention and usual care sites. We performed adjusted analyses to address the differences in demographics and baseline patient characteristics but there is potential for residual confounding. Second, sites were recruited from 6 health systems, which may limit broader generalizability to all practice settings. In addition, due to the combination of a limited number of sites and patients, the study was not powered enough to assess individual components of the primary and secondary outcomes. Third, the study had a relatively short follow-up period, which limited ability to assess longer term lipid optimization and clinical outcomes such as major adverse cardiovascular events. Finally, it is often challenging to control for or to collect data to cover the full range of possible influences with behavioral change interventions (such as clinician inertia). Although excluding the 178 enrolled patients without follow-up data from the primary analysis may bias the estimated treatment effect if loss to follow-up was differential by arm, we found similar attrition for both arms. And as shown in the intention-to-treat sensitivity analysis for primary endpoint, the results were similar. Also, because intact sites rather than individual patients were assigned to study arms, assignment was not randomized, and only six clusters were available, residual confounding from the marked baseline imbalance cannot be excluded despite prespecified adjusted analysis. For these reasons, between-arm comparisons should be considered quasi-experimental and hypothesis-generating rather than confirmatory.

## 5 Conclusions

In this quasi-experimental, cluster-assigned implementation trial, the multi-faceted intervention did not improve the primary composite lipid-optimization endpoint at one year. Participating intervention sites did achieve greater LDL-C reduction and higher rates of LDL-C goal attainment, and exploratory time-varying analyses suggested a possible delayed benefit; however, these findings are hypothesis-generating. Taken together with the baseline imbalance and the limited clinician engagement observed, these results suggest that realizing the potential of dashboard-based implementation strategies will require stronger EHR integration, sustained engagement mechanisms, and adequately powered randomized or larger cluster designs with longer follow-up to determine whether such programs improve LDL-C management among patients with ASCVD in the US.

## Funding

VICTORION-NOVEL was sponsored by Novartis Pharmaceuticals Corp.

## Ethical review statement

This study was approved by the Advarra IRB and IRB-approved informed consent forms were signed by clinician participants.

## Disclosures

MV has received research grant support, served on advisory boards, or had speaker engagements with Alnylam Pharmaceuticals, American Regent, Amgen, AstraZeneca, Bayer AG, Baxter Healthcare, BMS, Boehringer Ingelheim, Chiesi, Cytokinetics, Esperion, Fresenius Medical Care, Idorsia Pharmaceuticals, Lexicon Pharmaceuticals, Merck, Milestone Pharmaceuticals, Novartis, Novo Nordisk, Pharmacosmos, Recordati, Relypsa, Roche Diagnostics, Sanofi, and Tricog Health, and participates on clinical trial committees for studies sponsored by Amgen, AstraZeneca, Bayer AG, Boehringer Ingelheim, Galmed, Impulse Dynamics, Novartis, Occlutech, and Pharmacosmos. BD is an employee of Novartis Pharmaceuticals Corp. RM and NR worked on the study as employees of Premier Inc.

## Author agreement

All authors have reviewed and approved the final version of this manuscript and consent to its submission to the journal. The authors warrant that this work is original, has not been published previously (in whole or in part), and is not currently under consideration for publication elsewhere.

**Figure:**
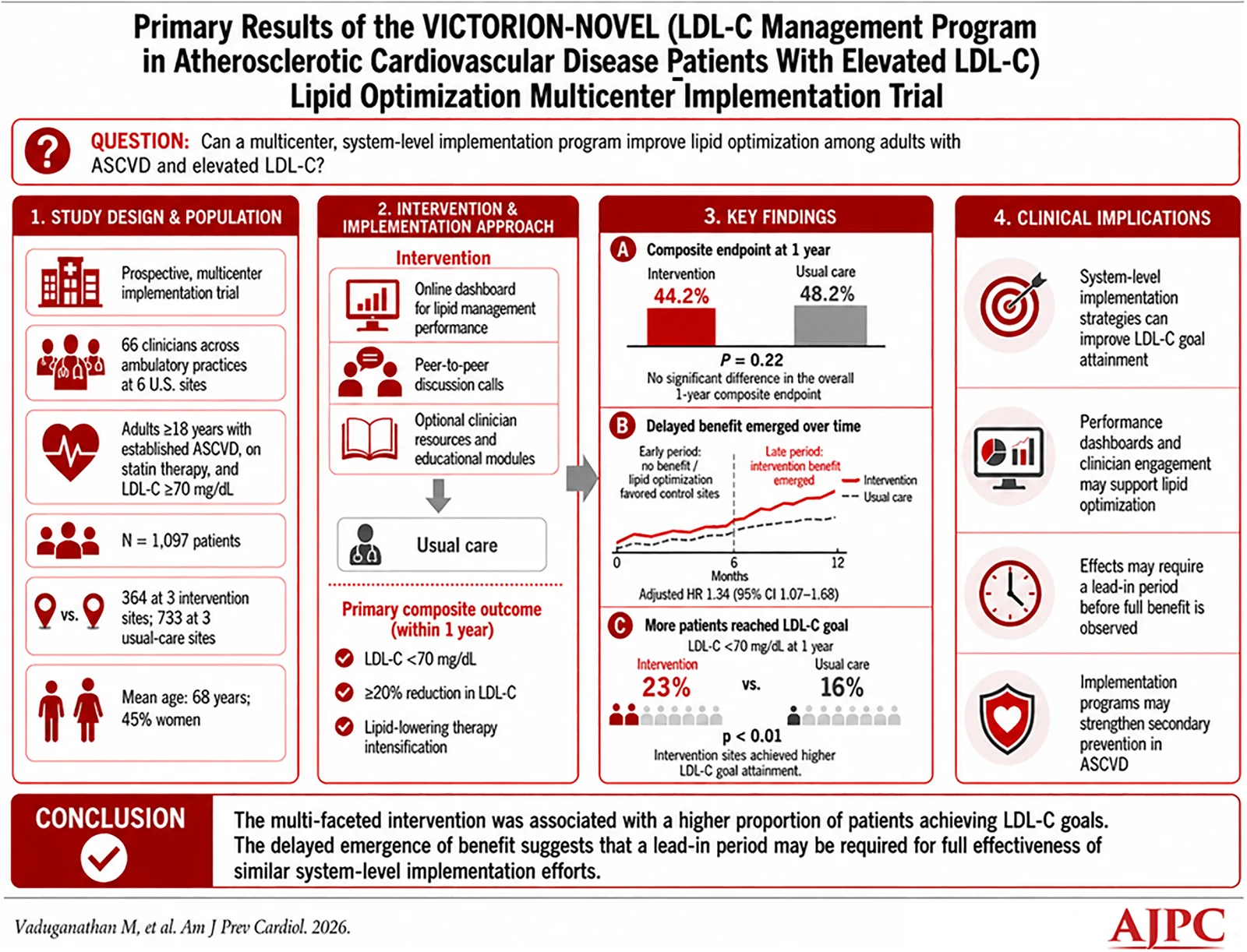

## CRediT authorship contribution statement

**Muthiah Vaduganathan:** Writing – review & editing, Supervision, Conceptualization. **Alan Brown:** Writing – review & editing, Conceptualization. **Stacy L. Farr:** Writing – review & editing, Conceptualization. **Rena Moon:** Writing – review & editing, Writing – original draft, Methodology, Formal analysis, Data curation. **Ning Rosenthal:** Writing – review & editing, Validation, Supervision, Methodology. **Batul Dahodwala:** Writing – review & editing, Methodology, Funding acquisition, Conceptualization. **Michael D. Shapiro:** Writing – review & editing, Supervision, Conceptualization.

## Declaration of competing interest

The authors declare the following financial interests/personal relationships which may be considered as potential competing interests: Rena Moon reports financial support was provided by Novartis Pharma AG. Muthiah Vaduganathan reports a relationship with Alnylam Pharmaceuticals Inc that includes: board membership, consulting or advisory, and funding grants. Muthiah Vaduganathan reports a relationship with American Regent Inc that includes: board membership, consulting or advisory, and funding grants. Muthiah Vaduganathan reports a relationship with Amgen Inc that includes: board membership, consulting or advisory, and funding grants. Muthiah Vaduganathan reports a relationship with AstraZeneca PLC that includes: board membership, consulting or advisory, and funding grants. Muthiah Vaduganathan reports a relationship with Bayer AG that includes: board membership, consulting or advisory, and funding grants. Muthiah Vaduganathan reports a relationship with Baxter Healthcare that includes: board membership, consulting or advisory, and funding grants. Muthiah Vaduganathan reports a relationship with Bristol-Myers Squibb Company that includes: board membership, consulting or advisory, and funding grants. Muthiah Vaduganathan reports a relationship with Boehringer Ingelheim Corp USA that includes: board membership, consulting or advisory, and funding grants. Muthiah Vaduganathan reports a relationship with Chiesi Pharmaceuticals GMBH that includes: board membership, consulting or advisory, and funding grants. Muthiah Vaduganathan reports a relationship with Cytokinetics Inc that includes: board membership, consulting or advisory, and funding grants. Muthiah Vaduganathan reports a relationship with Esperion Therapeutics Inc that includes: board membership, consulting or advisory, and funding grants. Muthiah Vaduganathan reports a relationship with Fresenius Medical Care AG that includes: board membership, consulting or advisory, and funding grants. Muthiah Vaduganathan reports a relationship with Idorsia Pharmaceuticals Ltd that includes: board membership, consulting or advisory, and funding grants. Muthiah Vaduganathan reports a relationship with Lexicon Pharmaceuticals Inc that includes: board membership, consulting or advisory, and funding grants. Muthiah Vaduganathan reports a relationship with Merck & Co Inc that includes: board membership, consulting or advisory, and funding grants. Muthiah Vaduganathan reports a relationship with Milestone Pharmaceuticals USA Inc that includes: board membership, consulting or advisory, and funding grants. Muthiah Vaduganathan reports a relationship with Novartis Pharmaceuticals Corporation that includes: board membership, consulting or advisory, and funding grants. Muthiah Vaduganathan reports a relationship with Novo Nordisk Inc that includes: board membership, consulting or advisory, and funding grants. Muthiah Vaduganathan reports a relationship with Pharmacosmos, Inc. that includes: board membership, consulting or advisory, and funding grants. Muthiah Vaduganathan reports a relationship with Recordati AG that includes: board membership, consulting or advisory, and funding grants. Muthiah Vaduganathan reports a relationship with Relypsa that includes: board membership, consulting or advisory, and funding grants. Muthiah Vaduganathan reports a relationship with Roche Diagnostics Corporation that includes: board membership, consulting or advisory, and funding grants. Muthiah Vaduganathan reports a relationship with Sanofi that includes: board membership, consulting or advisory, and funding grants. Muthiah Vaduganathan reports a relationship with Tricog Health that includes: board membership, consulting or advisory, and funding grants. Batul Dahodwala worked on the study as an employee of Novartis. Rena Moon and Ning Rosenthal worked on the study as employees of Premier Inc. If there are other authors, they declare that they have no known competing financial interests or personal relationships that could have appeared to influence the work reported in this paper.

## Data Availability

The data that support the findings of this study were originated by and are the property of Premier Inc. Requests for data sharing by license or by permission for the specific purpose of replicating results in this manuscript can be submitted to the Premier team at https://premierinc.com/real-world-evidence/real-world-data.
