# Supplementary material for: Primary results of the VICTORION-NOVEL (LDL-C management Program in atherosclerotic cardiovascular disease patients with elevated LDL-C) lipid optimization multicenter implementation trial
Source: Am J Prev Cardiol. 2026 Jul 16;30:101748. doi: 10.1016/j.ajpc.2026.101748 (PMC13633895; doi:10.1016/j.ajpc.2026.101748)
Supplement: Supplementary file 1 [file mmc1.docx]

**Supplementary Materials**

**Primary Results of the VICTORION-NOVEL (LDL-C management Program in Atherosclerotic Cardiovascular Disease patients with Elevated LDL-C) Lipid Optimization Multicenter Implementation Trial**

Muthiah Vaduganathan, MD, MPH; Alan Brown, MD; Stacy L Farr, PhD, MPH; Rena Moon, MD, MPH; Ning Rosenthal, MD, PhD, MPH; Batul Dahodwala, PhD; Michael D. Shapiro, MD

Table S1	…………………………………………………………………………………..	page 2

Table S2	…………………………………………………………………………………..	page 4

Table S3	…………………………………………………………………………………..	page 5

Figure S1	…………………………………………………………………………………..	page 6

Figure S2	…………………………………………………………………………………..	page 8

Figure S3	…………………………………………………………………………………..	page 9

**Table S1.** Baseline demographics and clinical characteristics of patients included in the analysis vs. patients lost to follow-up

|  | **Overall** | | **Included** | | **Excluded** | | **p-values** |
| --- | --- | --- | --- | --- | --- | --- | --- |
|  | **(N = 1,275)** | | **(N = 1,097)** | | **(N = 178)** | |  |
| **Age Category, n(%)** |  |  |  |  |  |  |  |
| 18-39 years | 7 | 0.5% | 7 | 0.6% | 0 | 0.0% | 0.5101*^C^* |
| 40-59 years | 266 | 20.9% | 230 | 21.0% | 36 | 20.2% |  |
| 60-74 years | 625 | 49.0% | 542 | 49.4% | 83 | 46.6% |  |
| 75+ years | 377 | 29.6% | 318 | 29.0% | 59 | 33.1% |  |
| **Age, continuous, in years** |  |  |  |  |  |  |  |
| Mean-Std Dev | 68.00 | 11.01 | 67.83 | 10.97 | 69.03 | 11.21 | 0.183*^T^* |
| Median (Q1-Q3) | 68.00 | (61.00- 76.00) | 68.00 | (61.00- 76.00) | 69.50 | (61.00- 78.00) |  |
| **Female sex, n(%)** | 574 | 45.0% | 493 | 44.9% | 81 | 45.5% | 0.8883*^C^* |
| **Race, n(%)** |  |  |  |  |  |  |  |
| White | 1,088 | 85.3% | 937 | 85.4% | 151 | 84.8% | 0.8168*^C^* |
| Black | 94 | 7.4% | 79 | 7.2% | 15 | 8.4% |  |
| Other | 93 | 7.3% | 81 | 7.4% | 12 | 6.7% |  |
| **Ethnicity, n(%)** |  |  |  |  |  |  |  |
| Hispanic | 28 | 2.2% | 27 | 2.5% | 1 | 0.6% | 0.0031*^C^* |
| Non-Hispanic | 1,104 | 86.6% | 959 | 87.4% | 145 | 81.5% |  |
| Unknown | 143 | 11.2% | 111 | 10.1% | 32 | 18.0% |  |
| **Primary insurance payer, n(%)** |  |  |  |  |  |  |  |
| Medicare | 281 | 22.0% | 225 | 20.5% | 56 | 31.5% | 0.0197*^C^* |
| Medicaid | 10 | 0.8% | 8 | 0.7% | 2 | 1.1% |  |
| Commercial Insurance | 50 | 3.9% | 43 | 3.9% | 7 | 3.9% |  |
| Uninsured | 3 | 0.2% | 3 | 0.3% | 0 | 0.0% |  |
| Other/Unknown | 931 | 73.0% | 818 | 74.6% | 113 | 63.5% |  |
| **Hypertension, n(%)** | 707 | 55.5% | 639 | 58.2% | 68 | 38.2% | < 0.0001*^C^* |
| **Atrial fibrillation, n(%)** | 95 | 7.5% | 88 | 8.0% | 7 | 3.9% | 0.054*^C^* |
| **Familial hypercholesterolemia, n(%)** | 0 | 0.0% | 0 | 0.0% | 0 | 0.0% |  |
| **Baseline Charlson comorbidities, n(%)** |  |  |  |  |  |  |  |
| Myocardial infarction | 283 | 22.2% | 252 | 23.0% | 31 | 17.4% | 0.098*^C^* |
| Congestive heart failure | 200 | 15.7% | 181 | 16.5% | 19 | 10.7% | 0.0474*^C^* |
| Peripheral vascular disease | 327 | 25.6% | 270 | 24.6% | 57 | 32.0% | 0.0357*^C^* |
| Cerebrovascular disease | 480 | 37.6% | 417 | 38.0% | 63 | 35.4% | 0.5034*^C^* |
| Dementia | 8 | 0.6% | 7 | 0.6% | 1 | 0.6% | 0.9048*^C^* |
| Chronic pulmonary disease | 133 | 10.4% | 128 | 11.7% | 5 | 2.8% | 0.0003*^C^* |
| Rheumatic disease | 10 | 0.8% | 10 | 0.9% | 0 | 0.0% | 0.201*^C^* |
| Peptic ulcer disease | 7 | 0.5% | 6 | 0.5% | 1 | 0.6% | 0.9802*^C^* |
| Mild liver disease | 12 | 0.9% | 11 | 1.0% | 1 | 0.6% | 0.572*^C^* |
| Diabetes | 234 | 18.4% | 217 | 19.8% | 17 | 9.6% | 0.0011*^C^* |
| Hemiplegia or paraplegia | 6 | 0.5% | 5 | 0.5% | 1 | 0.6% | 0.848*^C^* |
| Renal disease | 126 | 9.9% | 118 | 10.8% | 8 | 4.5% | 0.0094*^C^* |
| Moderate or severe liver disease | 1 | 0.1% | 1 | 0.1% | 0 | 0.0% | 0.687*^C^* |
| Any malignancy | 52 | 4.1% | 51 | 4.6% | 1 | 0.6% | 0.0105*^C^* |
| Metastatic solid tumor | 1 | 0.1% | 1 | 0.1% | 0 | 0.0% | 0.687*^C^* |
| AIDS/HIV | 1 | 0.1% | 1 | 0.1% | 0 | 0.0% | 0.687*^C^* |
| **Charlson Comorbidity Index (CCI) category, n(%)** |  |  |  |  |  |  |  |
| 0 | 207 | 16.2% | 177 | 16.1% | 30 | 16.9% | 0.0033*^C^* |
| 1-3 | 954 | 74.8% | 810 | 73.8% | 144 | 80.9% |  |
| 4+ | 114 | 8.9% | 110 | 10.0% | 4 | 2.2% |  |
| **Mean CCI (std. dev.)** | 1.74 | 1.74 | 1.82 | 1.82 | 1.26 | 1.09 | < 0.005*^T^* |
| **Patients with an ASCVD event at baseline, n(%)** |  |  |  |  |  |  |  |
| Acute MI or other ischemic heart diseases | 313 | 24.5% | 276 | 25.2% | 37 | 20.8% | 0.34*^C^* |
| Ischemic stroke | 352 | 27.6% | 302 | 27.5% | 50 | 28.1% |  |
| TIA | 50 | 3.9% | 43 | 3.9% | 7 | 3.9% |  |
| PAD | 196 | 15.4% | 165 | 15.0% | 31 | 17.4% |  |
| Unstable angina | 12 | 0.9% | 12 | 1.1% | 0 | 0.0% |  |
| Revascularization procedure | 239 | 18.7% | 198 | 18.0% | 41 | 23.0% |  |
| Median (Q1-Q3) | 7.00 | (3.00- 10.00) | 7.00 | (3.00- 10.00) | 6.00 | (3.00- 10.00) |  |
| **LLT treatment regimen, n(%)** |  |  |  |  |  |  |  |
| Statin | 1,140 | 89.4% | 972 | 88.6% | 168 | 94.4% | 0.1951*^C^* |
| Ezetimibe | 0 | 0.0% | 0 | 0.0% | 0 | 0.0% |  |
| PCSK9i | 0 | 0.0% | 0 | 0.0% | 0 | 0.0% |  |
| Bempedoic acid | 0 | 0.0% | 0 | 0.0% | 0 | 0.0% |  |
| Inclisiran | 0 | 0.0% | 0 | 0.0% | 0 | 0.0% |  |
| Statins + ezetimibe | 107 | 8.4% | 98 | 8.9% | 9 | 5.1% |  |
| Statins + PCSK9i | 16 | 1.3% | 15 | 1.4% | 1 | 0.6% |  |
| Statins + bempedoic acid | 3 | 0.2% | 3 | 0.3% | 0 | 0.0% |  |
| Statins + inclisiran | 0 | 0.0% | 0 | 0.0% | 0 | 0.0% |  |
| Statin + 2 or more | 9 | 0.7% | 9 | 0.8% | 0 | 0.0% |  |

**Table S2.** The probability of experiencing a composite endpoint in the intention-to-treat analysis

|  | **Unadjusted** | | **Adjusted^a^** | |
| --- | --- | --- | --- | --- |
|  | **HR** | **95% CI** | **HR** | **95% CI** |
| **Probability of experiencing composite endpoint prior to post-index day 49** |  |  |  |  |
| Intervention | 0.87 | 0.45-1.66 | 0.73 | 0.52-1.02 |
| Control | 1.00 | Ref | 1.00 | Ref |
| **Probability of experiencing composite endpoint between post-index day 50-218** |  |  |  |  |
| Intervention | 0.64 | 0.35-1.20 | **0.54** | **0.35-0.82** |
| Control | 1.00 | Ref | 1.00 | Ref |
| **Probability of experiencing composite endpoint on or after post-index day 219** |  |  |  |  |
| Intervention | **1.66** | **1.08-2.55** | **1.33** | **1.08-1.63** |
| Control | 1.00 | Ref | 1.00 | Ref |

^a^Adjusted for race, ethnicity, and hypertension (significant covariates for the final model)

**Table S3.** Implementation and engagement metrics at intervention sites

| **Implementation and engagement metric** | **Value (intervention sites)** | **Notes / definition** |
| --- | --- | --- |
| Peer-to-peer (P2P) call attendance, clinicians | Call 1 – 40 (100%);  Call 2 – not available;  Call 3 – 15 (38%) | Three intervention sites combined  #1 November 2023 - Platform overview, framework discussion, and interactive function of tools  #2 April 2024 - Rapid review of current lipid management guidelines  #3 July 2024 - Participant feedback and reflection |
| Focus-group participation, clinicians | 13 (20%) total (6 / 3 / 4 across the three site calls) | One focus group per intervention site individually at the end of the study in September and October 2024. |
| Clinician survey completion | Baseline 66 (100%): 40 intervention / 26 control  Post-intervention 37 (56%): 14 intervention / 23 control | Pre- and post-intervention clinician surveys |

**Figure S1.** Online dashboard examples

**Figure S2.** Site, clinician, and patient-level CONSORT flow diagram

**Figure S3.** Components of the Primary Composite Lipid Optimization Endpoint

a. Inverse Kaplan-Meier curve for the probability of achieving LDL-C goal <70mg/dL

b. Inverse Kaplan-Meier curve for the probability of achieving 20% reduction in LDL-C

c. Inverse Kaplan-Meier curve for the probability of LLT intensification
